# Outcomes of critically ill COVID-19 patients boarding in the emergency department of a tertiary care center in a developing country: a retrospective cohort study

**DOI:** 10.1186/s12245-023-00551-8

**Published:** 2023-10-13

**Authors:** Tharwat El Zahran, Sally Al Hassan, Victoria Al Karaki, Lina Hammoud, Christelle El Helou, Malak Khalifeh, Moustafa Al Hariri, Hani Tamim, Imad El Majzoub

**Affiliations:** 1 Department of Emergency Medicine, American University of Beirut Medical Center, Beirut, Lebanon; 2 QU Health, Qatar University, Doha, Qatar; 3 Department of Internal Medicine, American University of Beirut Medical Center, Beirut, Lebanon; 4 College of Medicine, Alfaisal University, Riyadh, Saudi Arabia

**Keywords:** COVID-19, Boarders, Pandemic, Emergency department, Critically ill patients

## Abstract

**Background:**

Boarding of critically ill patients in the emergency department (ED) has long been known to compromise patient care and affect outcomes. During the COVID-19 pandemic, multiple hospitals worldwide experienced overcrowded emergency rooms. Large influx of patients outnumbered hospital beds and required prolonged length of stay (LOS) in the ED. Our aim was to assess the ED LOS effect on mortality and morbidity, in addition to the predictors of in-hospital mortality, intubation, and complications of critically ill COVID-19 ED boarder patients.

**Methods:**

This was a retrospective cohort study, investigating 145 COVID-19-positive adult patients who were critically ill, required intensive care unit (ICU), and boarded in the ED of a tertiary care center in Lebanon. Data on patients who boarded in the emergency from January 1, 2020, till January 31, 2021, was gathered and studied.

**Results:**

Overall, 66% of patients died, 60% required intubation, and 88% developed complications. Multiple risk factors were associated with mortality naming age above 65 years, vasopressor use, severe COVID pneumonia findings on CT chest, chemotherapy treatment in the previous year, cardiovascular diseases, chronic kidney diseases, prolonged ED LOS, and low SaO_2_ < 95% on triage. In addition, our study showed that staying long hours in the ED increased the risk of developing complications.

**Conclusion:**

To conclude, all efforts need to be drawn to re-establish mitigation strategies and models of critical care delivery in the ED to alleviate the burden of critical boarders during pandemics, thus decreasing morbidity and mortality rates. Lessons from this pandemic should raise concern for complications seen in ED ICU boarders and allow the promotion of health measures optimizing resource allocation in future pandemic crises.

## Background

The National Academy of Medicine in the USA considers overcrowded emergency departments (EDs) as a national epidemic [1]. One of the most pressing challenges in a mass influx scenario is the care of ED Boarders, specifically the intensive care unit (ICU) boarders. ED boarders are the most vulnerable population in the ED due to fragmented care, multiple rotating medical teams with varying experience, and the lack of a safe nursing staffing model. ED boarding also contributes to low-quality process-related care of the critically ill patients waiting for ICU admission and at the same time compromise care to regular patients coming to the ED [2]. ED boarding of ill patients is associated not only with worse clinical outcomes but also with a huge financial burden [2, 3]. Boarding for more than 7 h is known to induce a longer duration of mechanical ventilation, an increase in the length of stay in the ICU, and more hospital-associated complications are usually seen with a significant rise of in-hospital mortality [2, 4–7]. Boarding patients have delays in-home medication administration, fluid boluses, antibiotic initiation, and disease-specific protocoled care. Furthermore, patients boarding in the ED have more medication-related adverse events than inpatients and impose both cognitive and emotional strains on emergency physicians [8]. Given this, many efforts were put into devising mitigation strategies and models of critical care delivery in the ED to alleviate the burden of ICU boarders [8].

Despite numerous efforts to counter this problem, the COVID-19 pandemic imposed a great strain on healthcare systems all over the world; thus, the crisis of overcrowded EDs with boarders resurfaced. During the period of August 1, 2020, to December 4, 2021, the number of COVID-19-associated hospitalizations in the USA reached 3,447,499 with up to 37.5% of COVID-19 hospitalized patients requiring admission to the critical care unit [9]. In Lebanon, the first COVID-19 case reported was on February 21, 2020 [10]. From that day till December 4, 2021, the cumulative number of COVID-19 cases had reached 677,147 with an ICU occupancy rate of 72% all over the country [11]. This substantial volume of patients has overwhelmed the capacities of many hospitals, leading to overcrowding and prolonged stays of boarding ICU patients in the ED [12]. In Lebanon, most of the hospitals are private healthcare facilities whereas the public hospitals compose only around 15% of a total number of hospital beds in the country [13]. Note that these public hospitals were mostly not well equipped, understaffed, and underfunded [13]. This is why the early pandemic was characterized by limited engagement of private hospitals, under equipment of public hospitals along with a deficit in COVID-19 units and ICU beds [13]. In parallel, even In Europe, emergency rooms were overwhelmed with patients and noted a decline in access to care for regular patients [14]. The ED at the American University of Beirut Medical Center (AUBMC) was recovering from the Beirut Port Explosion (BPE) when COVID-19 cases started to rise significantly which added a strain to its already limited resources and capacity. A study by Fares et al. showed that the BPE resulted in a significant increase in the daily number of positive COVID-19 cases and hospitalized COVID ICU patients [15]. The mass influx of COVID ICU boarders in our ED overwhelmed our existing resources and the surge required the activation of a disaster plan [15]. While many studies reported on the outcome of COVID ICU patients who got admitted to ICUs, no studies investigated COVID ICU patients boarding in the ED except one study by Tuttle et al., who assessed all critically ill patients boarding in the ED and not specifically COVID-19 patients [16]. Their study findings showed that hospital mortality was not significantly increased in ICU-bound patients with COVID-19 infections [16]. Our study assessed predictors of in-hospital mortality, intubation, and complications of critically ill COVID-19 patients boarding in the ED at a tertiary care center in Lebanon and looked into the repercussions of the length of stay in terms of developing complications and the rate of in-hospital mortality.

## Methods

### Study design and setting

This was a retrospective cohort study, single-centered at the AUBMC, conducted between January 1, 2020, and January 31, 2021. The AUBMC is the largest tertiary care center in Lebanon. The center has 358 beds and receives approximately 55,000 ED visits and approximately 25,000 inpatient admissions annually. Pediatric patients comprise 20% of the ED visits and 17% of hospital admissions. Most ED patients (75%) are covered through private insurance, whereas 23% pay out of pocket, and 2% are covered through governmental insurance. The Institutional Review Board at AUBMC approved the conduction of the study under IRB-ID BIO-2021–0021.

### Selection of participants

Adult patients (^3^18 years of age) presenting to the ED with COVID-19 infection were identified through the electronic health system (Epic Systems, Verona, WI, USA). The study sample included patients who were flagged as critically ill COVID-19 patients, requiring ICU care, and boarded in the ED. It compiled data of every patient who was confirmed to be COVID-19 positive through a positive PCR test or CT findings of COVID-19 infection and met at least one of the following criteria (admission to the COVID-ICU unit): (a) the 1st admission request patient service was to an ICU setting, (b) the last admission request was to an ICU setting, (c) ED disposition as “dead in ED,” or (d) ED patient who was placed on mechanical ventilation, face mask above 5L, non-rebreather face mask above 5L, high-flow nasal cannula, or BiPAP. These criteria ensured the maximum sensitivity in capturing all the COVID ICU ED boarding patients.

Patients who were initially admitted to a regular floor without boarding in the ED and during their stay on the floor required direct admission to ICU units were excluded from the study since our interest was in those boarding in the ED. In addition, patients who did not require ICU care ever and those who did not board in the ED were excluded. Additionally, an ICU patient was considered as a boarder if they remained in the ED for more than 5 h after receiving an ICU admission order, as per our ED policy that is based on an agreement between the departments of the AUBMC.

### Data collection and measure definitions

The study assessed the characteristics of COVID-19-infected patients in Lebanon who presented to the ED and required admission to the ICU but boarded in the ED due to a lack of bed availability. In-hospital mortality, intubation, and complication rates in this subset group of patients were noted. All patient’s information was filled into REDCap, a secure web-based application designed to support data capture for research studies that is Health Insurance Portability and Accountability Act compliant [17]. The data gathered info on patient’s demographics (age and gender), lifestyle (history of smoking), past medical history and surgical history within 1 month, ICU admission reasons, ED disposition, ED and ICU length of stay, and ED and hospital treatment and management. We also documented all medications administered, laboratory workup, and cultures done while in the ED. A section was designed to document the treatment provided (e.g., vasopressors, oxygen supplementation, intubation), the receipt of blood products (blood, plasma, or platelets), and all procedures performed (e.g., chest X-ray, CT scans, central line insertions, dialysis, embolization, chest tube placement).

The ED length of stay (LOS) was measured by calculating the time difference between the date and time of disposition and the date and time upon presentation to ED. For patients who were admitted to ICU, the date of admission to ICU was considered the disposition date from ED. To note, the disposition date applied is when the patient physically left the ED, not when the transfer order was placed on the system. In addition, for patients who died in ED, the death date was used as the disposition date from the ED. The primary dependent variable was mortality. Other dependent variables were assessed including endotracheal intubation or other health complications.

### Statistical analysis

Statistical analysis was performed using SPSS version 25.0 (Armonk, NY: IBM Corp) statistical package. Descriptive analysis was conducted by calculating frequencies and percentages for categorical variables, whereas for continuous variables, means, standard deviations, ranges, and percentiles were used. Bivariate analysis was performed using chi-square and Student’s *T* tests to examine associations between different variables and dependent variables when appropriate. The primary dependent variable was mortality. Other dependent variables were assessed including endotracheal intubation or health complications.

Logistic regression was performed to identify independent predictors for mortality. Variables that were found to be significant at the bivariate level were selected for inclusion in the multivariate regression analysis. More specifically, forward stepwise logistic regression analysis was used, where odds ratio (OR) and 95% confidence interval (CI) were presented. The time of death for the patient who died and the hospital length of stay for the patient who survived during the hospital stay were used as time variables for constructing the Kaplan–Meier curves (KM). Logrank test was used to assess the statistical significance of the KMs. Statistical significance was considered at a *p* value ≤ 0.05.

## Results

### Demographics and clinical characteristics of COVID ICU patients

A total of 145 COVID ICU patients were enrolled in the study. The average age was 67.6 ± 14.3 years, range [18–96] years, and among them, 103 (71.0%) patients were males and 59 (43.7%) were smokers. The participants’ top three most common comorbid conditions were hypertension (*n* = 91, 62.8%), diabetes mellitus (*n* = 62, 42.8%), and cardiovascular diseases (*n* = 62, 42.8%) (Table 1). Most COVID ICU patients had low oxygen saturation levels < 95% at triage (*n* = 101, 70.1%). The average ED boarding time was 6.8 days ± 4.9. In total, 95 (65.5%) died, among them, 52 (55.0%) died in ICU and the remaining 42 (45.0%) died in ED (Table 1). A cutoff age value of 65.5 years (AUC = 0.665, 95% CI 0.569–0.761, sensitivity = 0.705, 1-specificity = 0.46) was used for analysis. To note, an average of ED length of stay of around 7 days was found (*p* value 0.03).

**Table 1:** Association of demographic and clinical characteristics of COVID ICU patients with in-hospital mortality

**Characteristics**		**Total*, n* = 145**	**Alive, *n* = 50 (34.5%)**	**Dead, *n* = 95 (65.5%)**	***p* value**
**Age (years)**	** ≤ 65**	55 (37.9%)	27 (54%)	28 (29.5%)	**0.004**
	** > 65**	90 (62.1%)	23 (46%)	67 (70.5%)	
**Sex**	**Female**	42 (29%)	15 (30%)	27 (28.4%)	0.842
	**Male**	103 (71%)	35 (70%)	68 (71.6%)	
**Smoking Status**	**Non-smoker**	76 (56.3%)	26 (56.5%)	50 (56.2%)	0.97
	**Smoker**	59 (43.7%)	20 (43.5%)	39 (43.8%)	
**BMT within 1 year**		4 (16%)	1 (14.3%)	3 (16.7%)	1.000
**Chemotherapy within 1 year**		11 (44%)	2 (28.6%)	9 (50%)	0.407
**ED LOS (days)**		6.80 ± 4.9	5.58 ± 0.49	7.45 ± 0.67	**0.03**
**Comorbidities**	**Cardiovascular Diseases**	62 (42.8%)	18 (36%)	44 (46.3%)	0.233
	**Diabetes mellitus**	62 (42.8%)	21 (42%)	41 (43.2%)	0.893
	**Hypertension**	91 (62.8%)	30 (60%)	61 (64.2%)	0.618
	**Dyslipidemia**	37 (25.5%)	10 (20%)	27 (28.4%)	0.269
	**Cerebrovascular accident/transient ischemic attack**	8 (5.5%)	2 (4%)	6 (6.3%)	0.715
	**Chronic obstructive Pulmonary disease**	9 (6.2%)	4 (8%)	5 (5.3%)	0.496
	**Chronic kidney disease**	23 (15.9%)	4 (8%)	19 (20%)	0.060
	**Liver disease**	2 (1.4%)	1 (2%)	1 (1.1%)	1.000

*BMT* bone marrow transplant, *LOS* length of stay

### Treatments and health-related complications of COVID ICU patients

The patients’ hospital treatment included steroids (*n* = 139, 95.9%), antibiotics (*n* = 137, 94.5%), anticoagulants and antiplatelets (*n* = 115, 79.3%), Remdesivir (*n* = 82, 56.6%), Actemra (*n* = 50, 34.5%), convalescent plasma (*n* = 50, 34.5%), Ivermectin (*n* = 48, 33.1%), Baricitinib (*n* = 23, 15.9%), and hydroxychloroquine (*n* = 3, 2.1%). About 58 patients (40.0%) received vasopressors (Table 2, in the tables’ section).

**Table 2:** Association of treatments and health-related complications of COVID ICU patients with in-hospital mortality

	**Options**	**Total, *n* = 145**	**Alive,* n* = 50 (34.5%)**	**Dead, *n* = 95 (65.5%)**	***p* value**
**Laboratory workup**	**ABGs pH**	7.4 ± 0.1	7.4 ± 0.1	7.4 ± 0.1	0.266
	**ABGs HCO**_**3**_** (mmol/L)**	20.6 ± 4.9	21.4 ± 4.7	20.1 ± 5	0.137
	**ABGs PCO**_**2**_** (mmHg)**	32.1 ± 7.2	32.8 ± 7.5	31.7 ± 7.1	0.400
	**ANC (10**^**3**^**- cu.mm)**	8.1 ± 5.1	8.0 ± 4.4	8.1 ± 5.5	0.936
	**Hemoglobin (g/dl)**	12.4 ± 2.3	13 ± 2	12.1 ± 2.3	**0.021**
	**Platelet count (10**^**3**^**- cu.mm)**	234.5 ± 124.7	245.3 ± 116.5	228.7 ± 129.2	0.453
	**Creatinine (mg/dl)**	1.4 ± 1.4	1.3 ± 0.9	1.6 ± 1.5	0.223
	**D-Dimer (10**^**3**^**- ng/ml)**	1.7 ± 3.9	1.2 ± 1.4	2.0 ± 4.7	0.288
	**Fibrinogen (g/dl)**	5.7 ± 2	5.4 ± 1.8	5.8 ± 2	0.408
	**Troponin (ng/ml)**	0.049 ± 0.083	0.026 ± 0.029	0.058 ± 0.095	**0.008**
	**CRP (mg/l)**	165.6 ± 98.4	152.8 ± 95.8	171.8 ± 99.5	0.296
	**Procalcitonin (ng/ml)**	1.1 ± 2.4	0.5 ± 0.8	1.4 ± 2.8	**0.010**
	**PT (sec)**	16 ± 6.4	15.7 ± 7.4	16.2 ± 5.9	0.705
	**aPTT (sec)**	30.3 ± 6.4	29.9 ± 4.6	30.5 ± 7.1	0.627
	**INR**	1.4 ± 0.6	1.4 ± 0.7	1.4 ± 0.5	0.740
	**WBC (10**^**3**^**- cu.mm)**	9.8 ± 8.0	8.9 ± 4.7	10.3 ± 9.3	0.329
**Hospital treatment**	**Steroids**	139 (95.9%)	48 (96%)	91 (95.8%)	1.000
	**Anticoagulants and antiplatelets**	115 (79.3%)	38 (76%)	77 (81.1%)	0.475
	**Antibiotics/antifungals/antivirals**	137 (94.5%)	44 (88%)	93 (97.9%)	**0.020**
	**Remdesivir**	82 (56.6%)	24 (48%)	58 (61.1%)	0.132
	**Baricitinib**	23 (15.9%)	9 (18%)	14 (14.7%)	0.609
	**Actemra**	50 (34.5%)	24 (48%)	26 (27.4%)	**0.013**
	**Ivermectin**	48 (33.1%)	16 (32%)	32 (33.7%)	0.838
	**Convalescent plasma**	50 (34.5%)	13 (26%)	37 (38.9%)	0.119
**Complications**	**Respiratory complications** ^**a**^	89 (61.4%)	17 (34%)	72 (75.8%)	** < 0.001**
	**ARDS**	58 (40%)	11 (22%)	47 (49.5%)	**0.001**
	**Pulmonary embolism**	17 (11.7%)	7 (14%)	10 (10.5%)	0.537
	**Pneumothorax or pneumomediastinum**	19 (13.1%)	2 (4%)	17 (17.9%)	**0.018**
	**Respiratory failure**	60 (41.4%)	10 (20%)	50 (52.6%)	** < 0.001**
	**AKI**	49 (33.8%)	7 (14%)	42 (44.2%)	** < 0.001**
	**Septic shock and DIC**	49 (33.8%)	6 (12%)	43 (45.3%)	** < 0.001**
	**Stroke or DVT**	6 (4.1%)	2 (4%)	4 (4.2%)	1.000
	**Cardiovascular**	71 (49%)	9 (18%)	62 (65.3%)	** < 0.001**
	**Infection**	34 (23.4%)	10 (20%)	24 (25.3%)	0.477
	**Others** ^**b**^	18 (12.4%)	0 (%)	18 (18.9%)	**0.001**
	**Dialysis**	23 (15.9%)	6 (12%)	17 (17.9%)	0.356
**O**_**2**_** requirement**	**BIPAP**	56 (38.6%)	17 (34%)	39 (41.1%)	0.407
	**O**_**2**_** high flow**	22 (15.2%)	4 (8%)	18 (18.9%)	0.081
	**Intubation**	93 (64.1%)	18 (36%)	75 (78.9%)	** < 0.001**

^a ^Respiratory complications include ARDS, pneumothorax, respiratory failure, or pneumomediastinum

^b^ Other complications are metabolic acidosis or hemorrhage or cecal perforation or rhabdomyolysis

About 88.3% of COVID ICU patients developed health-related complications (*n* = 128). Renal failure (*n* = 85, 57.9%) was the most commonly reported complication followed by cardiovascular complications (*n* = 71, 49.0%), acute respiratory distress syndrome (*n* = 58, 40.0%), septic shock and DIC (*n* = 49, 33.8%), infection (*n* = 34, 23.4%), and pneumothorax (*n* = 19, 13.1%) (Table 2). About 23 patients (15.9%) underwent dialysis and only 5 patients had a tracheostomy inserted. Oxygen was provided to COVID ICU patients through different techniques; BiPAP (*n* = 56, 38.6%) high-flow nasal cannula (*n* = 22, 15.2%) or endotracheal intubation (*n* = 87, 60.0%) (Table 2). Interestingly, out of patients who developed pneumothorax, a trend showed that 16 cases (17.2%) required intubation whereas 3 cases (5.8%) did not, with an exact *p* value of 0.05.

### Predictors of In-Hospital Mortality in COVID ICU patients

As summarized in Table 1, 71.6% of patients were males (*n* = 68, *p* = 0.842). It also showed that they were significantly older (70.5% vs. 46.0%, *p* = 0.004). They were more likely to have had chemotherapy in the previous year (50% (*n* = 9) vs 28.6% (*n* = 2), *p* = 0.407). Mortality was significantly associated with ED LOS, as our analysis showed that patients who died stayed longer in the ED compared to those who did not die (7.45 days versus 5.58 days, *p* = 0.03) (Table 1). However, there was no significant difference in terms of gender, smoking status, and types of comorbidities between COVID ICU patients who died or survived. Patients who died were more likely to have cardiovascular diseases (46.3% vs. 36.0%) and 2.9 times more likely to have chronic kidney diseases (20.0% vs. 8.0%), but it was not statistically significant (*p* > 0.05) (Table 1). Patients who died were twice more likely to have low oxygen saturation levels < 95% at triage (75.5% vs. 60.0%, *p* = 0.053) and 1.9 times more likely to have severe COVID disease on CT (60% vs. 44%, *p* = 0.066) but was not significant. They significantly required 4.9 times more vasopressors (54.7% vs. 12.0%, *p* < 0.001). Intubated patients were 6.7 times significantly more prone to death (78.9% vs. 36.0%, *p* < 0.001) (Table 2).

Treatment with convalescent plasma, steroids, anticoagulants, Remdesivir, or Ivermectin did not significantly affect mortality (*p* > 0.05) (Table 2). However, patients in the hospital who received Actemra were significantly less likely to die than those who did not receive Actemra (27.4% vs. 48%, *p* = 0.013).

Patients who died developed multiple complications including acute respiratory distress syndrome (ARDS) (49.5% vs. 22.0%, *p* = 0.001), pneumothorax (17.9% vs. 4.0%, *p* = 0.018), acute kidney injury (44.2% vs. 14.0%, *p* < 0.001), respiratory failure (52.6% vs. 20.0%, *p* < 0.001), septic shock and DIC (45.3% vs. 12.0%, *p* > 0.001), and cardiovascular complications (65.3% vs. 18.0%, *p* < 0.001) (Table 2).

Additionally, 41.1% and 18.9% of patients who were on BiPAP, and high-flow nasal cannula died, respectively. There was no significant difference between patients who were on BiPAP and died versus those who survived. Similarly, there was no significant difference between patients who were on high-flow nasal cannula and died versus those who survived (Table 2).

However, almost 78.9% of patients who got intubated have died (78.9% vs. 36.0%, *p* < 0.001) (Table 2). There was no significant difference in the ED boarding time between patients who died or survived. Half of the patients who died stayed more than 6 days in the ED (51.9%).

Additionally, 41.1% and 18.9% of patients who were on BiPAP and high-flow nasal cannula died, respectively. There was no significant difference between patients who survived or not and were on BiPAP or high-flow nasal cannula (Table 2).

For the laboratory results, troponin (0.058 ± 0.095 vs 0.026 ± 0.029, *p* = 0.008) and procalcitonin (1.4 ± 2.8 vs. 0.5 ± 0.8, *p* = 0.01) were significantly higher in patients who died than those who survived. Hemoglobin level (12.1 ± 2.3 vs. 13.0 ± 2.0, *p* = 0.021) was significantly lower in patients who died. Other laboratory values including D-dimer, Fibrinogen, ANC, CRP, PT, WBC, and creatinine levels did not show a significant difference (Table 2).

### Predictors of mortality using logistic regression

After adjusting for confounding variables using logistic regression, patients > 65 years old were more likely to die (aOR = 11.66, 95%CI = 1.59–85.82). Patients who died were more likely to be on vasopressors (aOR = 11.56, 95%CI = 1.44–92.84) and to have a severe COVID-19 on CT (aOR = 7.07, 95%CI = 1.34–37.42). The patients who received Actemra in the ED had lower odds of dying (aOR = 0.08, 95%CI = 0.02–0.43). Patients who died had significantly more complications including pneumothorax (aOR = 20.7, 95%CI = 1.18–364.28), or cardiovascular complications (aOR = 10.33, 95%CI = 2.05–52.11). Mortality was more likely in those who received high-flow oxygen (aOR = 10.43, 95%CI = 1.14–95.66) and in patients who had endotracheal intubation (aOR = 5.79, 95%CI = 1.23–27.17) (Table 3).

**Table 3:** Logistic regression: factors associated with mortality in COVID ICU patients

	**aOR**	**95% C.I**	***p* value**
**Pneumothorax or pneumomediastinum**	20.77	[1.18, 364.28]	**0.038**
**Age (> 65 years)**	11.66	[1.59, 85.82]	**0.016**
**Vasopressors**	11.56	[1.44, 92.84]	**0.021**
**Highflow**	10.43	[1.14, 95.66]	**0.038**
**Cardiovascular complications**	10.33	[2.05, 52.11]	**0.005**
**Severe CT**	7.07	[1.34, 37.42]	**0.021**
**Intubation**	5.79	[1.23, 27.17]	**0.026**
**Actemra**	0.08	[0.02, 0.43]	**0.003**

Variables entered in the model: age (reference: ≤ 65 years old), O_2_ triage (reference ≥ 95%), kidney disease (reference: no), vasopressors (reference: no), severe CT (reference: no), ARDS (reference: no), pneumothorax or pneumomediastinum (reference: no), acute kidney injury (reference: no), septic shock or DIC (reference: no), cardiovascular complications (reference: no), Actemra (reference: no), Remdesivir (reference: no), convalescent plasma (reference: no), antibiotics/antifungals/antivirals (reference: no), highflow (reference: no), intubation (reference: no), hemoglobin level, ABGs HCO_3_, Troponin, and Procalcitonin

Omnibus test < 0.001, *R*^2^ = 0.701, Hosmer = 0.882. Hyper-coagulopathy includes stroke, deep venous thrombosis (DVT), or pulmonary embolism (PE). Respiratory complications include ARDS, pneumothorax, respiratory failure, or pneumomediastinum. Other complications are metabolic acidosis, hemorrhage or cecal perforation, or rhabdomyolysis. Abbreviations: *aOR* adjusted odds ratio, *95%CI* 95% confidence interval

### Predictors of intubation in COVID ICU patients

Of the total of 93 patients who were intubated, 70 (75.3%) were males, 61 (65.6%) patients were older than 65 years, and 76 (81.7%) patients had cardiovascular diseases. However, there was no significant difference in gender, age, smoking status, or types of comorbidities between COVID ICU patients who were intubated and those patients who were not intubated. Most patients who were intubated had low oxygen saturation at triage compared to patients who were not intubated (77.4% vs. 56.9%, *p* = 0.01) (Table 4, in the tables’ section).

**Table 4:** Association of COVID ICU patients’ demographic and clinical characteristics with intubation

		**Total, *n* = 145**	**No intubation, *n* = 52 (35.9%)**	**Intubation, *n* = 93 (64.1%)**	***p* value**
**Sex**	**Female**	42 (29%)	19 (36.5%)	23 (24.7%)	0.133
	**Male**	103 (71%)	33 (63.5%)	70 (75.3%)	
**Age**	** ≤ 65**	55 (37.9%)	23 (44.2%)	32 (34.4%)	0.242
	** > 65**	90 (62.1%)	29 (55.8%)	61 (65.6%)	
**O**_**2**_** triage**	** < 95**	101 (70.1%)	29 (56.9%)	72 (77.4%)	**0.01**
	** ≥ 95**	43 (29.9%)	22 (43.1%)	21 (22.6%)	
**ED LOS (days)**		6.80 ± 4.9	5.88 ± 4.99	7.32 ± 4.76	0.09
** Comorbidities**	**Cardiovascular comorbid**	114 (78.6%)	38 (73.1%)	76 (81.7%)	0.223
	**CVA**	8 (5.5%)	3 (5.8%)	5 (5.4%)	1
	**Dementia**	6 (4.1%)	3 (5.8%)	3 (3.2%)	0.667
	**Liver disease**	2 (1.4%)	1 (1.9%)	1 (1.1%)	1
	**Kidney disease**	23 (15.9%)	7 (13.5%)	16 (17.2%)	0.554
	**DM**	62 (42.8%)	19 (36.5%)	43 (46.2%)	0.258
	**PVD**	2 (1.4%)	0 (0%)	2 (2.2%)	0.537
	**COPD**	9 (6.2%)	3 (5.8%)	6 (6.5%)	1
	**Other comorbidities**	64 (44.1%)	26 (50%)	38 (40.9%)	0.288
***Hospital medications***					
**Vasopressor**		58 (40%)	7 (13.5%)	51 (54.8%)	** < 0.001**
**Steroids**		139 (95.9%)	49 (94.2%)	90 (96.8%)	0.461
**Anticoagulants or antiplatelets**		115 (79.3%)	39 (75%)	76 (81.7%)	0.338
**Antibiotics/antifungals/antivirals**		137 (94.5%)	46 (88.5%)	91 (97.8%)	**0.025**
**Remdesivir**		82 (56.6%)	22 (42.3%)	60 (64.5%)	**0.01**
**Baricitinib**		23 (15.9%)	8 (15.4%)	15 (16.1%)	0.906
***Complications***					
**Respiratory complications**		89 (61.4%)	20 (38.5%)	69 (74.2%)	** < 0.001**
**Acute kidney injury**		49 (33.8%)	11 (21.2%)	38 (40.9%)	**0.016**
**Septic shock and DIC**		49 (33.8%)	6 (11.5%)	43 (46.2%)	** < 0.001**
**Hyper-coagulopathy**		21 (14.5%)	4 (7.7%)	17 (18.3%)	0.082
**Cardiovascular complications**		71 (49%)	18 (34.6%)	53 (57%)	**0.01**
**Infection**		34 (23.4%)	6 (11.5%)	28 (30.1%)	**0.011**
**Other complications**		18 (12.4%)	4 (7.7%)	14 (15.1%)	.197
**Dialysis**		23 (15.9%)	1 (1.9%)	22 (23.7%)	**0.001**
***O***_***2***_*** requirement***					
**O**_**2**_** high flow**		22 (15.2%)	8 (15.4%)	14 (15.1%)	0.958
**O**_**2**_** BIPAP**		56 (38.6%)	24 (46.2%)	32 (34.4%)	0.164
**Sex**	**Female**	42 (29%)	19 (36.5%)	23 (24.7%)	0.133
	**Male**	103 (71%)	33 (63.5%)	70 (75.3%)	
**Age**	** ≤ 65**	55 (37.9%)	23 (44.2%)	32 (34.4%)	0.242
	** > 65**	90 (62.1%)	29 (55.8%)	61 (65.6%)	
**O**_**2**_** triage**	** < 95**	101 (70.1%)	29 (56.9%)	72 (77.4%)	**0.01**
	** ≥ 95**	43 (29.9%)	22 (43.1%)	21 (22.6%)	
**ED LOS (days)**		6.80 ± 4.9	5.88 ± 4.99	7.32 ± 4.76	0.09
** Comorbidities**	**Cardiovascular comorbid**	114 (78.6%)	38 (73.1%)	76 (81.7%)	0.223
	**CVA**	8 (5.5%)	3 (5.8%)	5 (5.4%)	1
	**Dementia**	6 (4.1%)	3 (5.8%)	3 (3.2%)	0.667
	**Liver disease**	2 (1.4%)	1 (1.9%)	1 (1.1%)	1
	**Kidney disease**	23 (15.9%)	7 (13.5%)	16 (17.2%)	0.554
	**DM**	62 (42.8%)	19 (36.5%)	43 (46.2%)	0.258
	**PVD**	2 (1.4%)	0 (0%)	2 (2.2%)	0.537
	**COPD**	9 (6.2%)	3 (5.8%)	6 (6.5%)	1
	**Other comorbidities**	64 (44.1%)	26 (50%)	38 (40.9%)	0.288
***Hospital medications***					
**Vasopressor**		58 (40%)	7 (13.5%)	51 (54.8%)	** < 0.001**
**Steroids**		139 (95.9%)	49 (94.2%)	90 (96.8%)	0.461
**Anticoagulants or antiplatelets**		115 (79.3%)	39 (75%)	76 (81.7%)	0.338
**Antibiotics/antifungals/antivirals**		137 (94.5%)	46 (88.5%)	91 (97.8%)	**0.025**
**Remdesivir**		82 (56.6%)	22 (42.3%)	60 (64.5%)	**0.01**
**Baricitinib**		23 (15.9%)	8 (15.4%)	15 (16.1%)	0.906
***Complications***					
**Respiratory complications** ^**a**^		89 (61.4%)	20 (38.5%)	69 (74.2%)	** < 0.001**
**Acute kidney injury**		49 (33.8%)	11 (21.2%)	38 (40.9%)	**0.016**
**Septic shock and DIC**		49 (33.8%)	6 (11.5%)	43 (46.2%)	** < 0.001**
**Hyper-coagulopathy**		21 (14.5%)	4 (7.7%)	17 (18.3%)	0.082
**Cardiovascular complications**		71 (49%)	18 (34.6%)	53 (57%)	**0.01**
**Infection**		34 (23.4%)	6 (11.5%)	28 (30.1%)	**0.011**
**Other complications** ^**b**^		18 (12.4%)	4 (7.7%)	14 (15.1%)	.197
**Dialysis**		23 (15.9%)	1 (1.9%)	22 (23.7%)	**0.001**
***O***_***2***_*** requirement***					
**O**_**2**_** high flow**		22 (15.2%)	8 (15.4%)	14 (15.1%)	0.958
**O**_**2**_** BIPAP**		56 (38.6%)	24 (46.2%)	32 (34.4%)	0.164

*CVA* cerebral vascular accident, *DM* diabetes mellites, *PVD* peripheral vascular disease, *COPD* chronic obstructive pulmonary disease, *DIC* disseminated intravascular coagulation

^a^Respiratory complications include ARDS, pneumothorax, respiratory failure, or pneumomediastinum

^b^Other complications are metabolic acidosis or hemorrhage or cecal perforation or Rhabdomyolysis. Hyper-coagulopathy includes stroke, deep venous thrombosis (DVT), or pulmonary embolism (PE)

Treatment with steroids, anticoagulants, Baricitinib, Ivermectin, or Actemra had no significant effect on intubation. About 57.1% and 63.6% of patients who were on BIPAP and high-flow nasal cannula, respectively, were intubated, but it was not significant (*p* > 0.05) (Table 4).

Compared to non-intubated patients, intubated patients had significantly more respiratory complications (74.2% vs. 38.5%, *p* < 0.001), as well as septic shock (46.2% vs. 11.5%, *p* < 0.001) or cardiovascular complications (57% vs. 34.6%, *p* = 0.01). Additionally, 95.6% of patients on dialysis were intubated (23.7% vs. 1.9%, *p* = 0.001). Patients who were on antibiotics, Remdesivir, and convalescent plasma were significantly more intubated (Table 4).

After adjusting for confounding variables using logistic regression, SaO_2_ < 95% at triage (aOR = 5.2, 95%CI = 1.58–17.17), convalescent plasma (aOR = 4.09, 95%CI = 1.28–13.14), and respiratory complications (aOR = 7.89, 95%CI = 2.53–24.62) remained significantly associated with intubation. Patients on BiPAP were significantly less intubated (aOR = 0.27, 95%CI = 0.09–0.795). Patients on vasopressors (aOR = 8.27, 95%CI = 2.43–28.15) or dialysis (aOR = 31.87, 95%CI = 2.73–372.08) were significantly more intubated (Table 5).

**Table 5:** Logistic regression: predictors of intubation in COVID ICU patients

	**aOR**	**95% C.I**	***p* value**
**Dialysis**	31.87	2.73, 372.08	**0.006**
**Vasopressor**	8.27	2.43, 28.15	**0.001**
**Respiratory complications**	7.89	2.53, 24.62	** < 0.001**
**O**_**2**_** at triage (< 95%)**	5.20	1.58, 17.17	**0.007**
**Convalescent plasma**	4.09	1.28, 13.14	**0.018**
**O**_**2**_** BiPAP**	0.27	0.09, 0.795	**0.018**

Variables entered in the model: O_2_ at triage (reference: ≥ 95%), sex (reference: female), HR at triage (reference: ≤ 100), O_2_ BiPAP (reference: no), dyslipidemia (reference: no), vasopressor (reference: no), hyper-coagulopathy complications (reference: no), septic shock or DIC complications (reference: no), cardiovascular complications (reference: no), infection complications (reference: no), LOS hospital (days), AKI complications (reference: no), respiratory complications (reference: no), chest CT pneumonia (reference: no), other complications (reference: no), ivermectin (reference: no), convalescent plasma (reference: no), antibiotics/antifungals/antivirals (reference: no), Remdesivir (reference: no), and dialysis (reference: no)

Omnibus test < 0.001, *R*^2^ = 0.625, Hosmer = 0.527. Hyper-coagulopathy includes stroke, deep venous thrombosis (DVT), or pulmonary embolism (PE). Respiratory complications include ARDS, pneumothorax, respiratory failure, or pneumomediastinum. Other complications include metabolic acidosis, hemorrhage, cecal perforation, and rhabdomyolysis. Abbreviations: *aOR* adjusted odds ratio, *95%CI* 95% confidence interval

### Predictors of complications in COVID ICU patients

Forty-nine patients developed septic shock (33.8%) and 89 patients had respiratory complications (61.4%) including ARDS, pneumothorax, respiratory failure, or pneumomediastinum. Most of them were males (73.5%, 69.7%, respectively) and older than 65 years old (59.2%, 61.8%, respectively). However, none of the gender, age, or type of comorbidities were significantly associated as predictors for septic shock or respiratory complications (*p* > 0.05). Patients who developed any complication stayed longer in the ED than those who had no complications (4.55 days ± 6.20 versus 7.12 days ± 4.60, *p* = 0.04). Treatment with steroids, antibiotics, anticoagulants, Baricitinib, Actemra, or Remdesivir had no significant association with respiratory complications, or septic shock (*p* > 0.05). Patients on convalescent plasma significantly developed more septic shock (46.9% vs. 28.1%, *p* = 0.024) and respiratory complications (41.6% vs. 23.2%, *p* = 0.024). Similarly, patients on Ivermectin significantly developed more respiratory complications (44.9% vs. 14.3%, *p* < 0.001).

Of the 17 patients who had a pulmonary embolism (PE) (11.7%), 10 were males (58.8%). Interestingly, patients who developed pulmonary embolism were significantly younger than 65 years old (64.7% vs. 34.4%, *p* = 0.015). However, there was no significant difference in gender or type of comorbidities between patients with or without pulmonary embolism. Treatment with steroids, anticoagulants, convalescent plasma, Actemra, or Remdesivir had no significant effects on PE (*p* > 0.05). Patients on Ivermectin (64.7% vs. 28.9%, *p* = 0.003) or Baricitinib (35.3% vs. 13.3%, *p* = 0.031) had significantly more PEs than those not on Ivermectin or Baricitinib.

After adjusting for confounding variables using logistic regression, vasopressor use (aOR = 3.28, 95%CI = 5.35–32.94) and Ivermectin (aOR = 3.03, 95%CI = 1.17–7.86) remained significantly associated with septic shock. O_2_ high flow (aOR = 0.048, 95%CI = 1.02–22.28), Ivermectin (aOR = 3.57, 95%CI = 1.35–9.49), and intubation (aOR = 8.73, 95%CI = 3.36–22.695) remained significantly associated with respiratory complications. Ivermectin (aOR = 4.04, 95%CI = 1.3–12.55), Baricitinib (aOR = 3.59, 95%CI = 1.04–12.37), and severe CT (aOR = 4.84, 95%CI = 1.19–19.61) remained significantly associated with pulmonary embolism.

## Discussion

The main aim of our study was to assess the association between ED boarding time, in-hospital mortality, complications, and the risk of intubation. It has been long known that ED crowding is a potential threat to the quality of care [18]. Our study showed a high mortality rate since most of our patients were elderly and had multiple comorbidities, including hypertension, DM, CVD, CKD, and chronic pulmonary disease. Eighty-eight percent of patients developed complications mainly respiratory failure and dialysis, followed by CVD and ARDS, septic shock, and pneumothorax. Almost 66% of our patients did not survive. Mortality was ten times more likely in those who were on high-flow oxygen than patients who underwent endotracheal intubation. Treatment received by patients did not significantly affect mortality, except for patients who received Actemra. These patients were significantly less likely to die. Troponin and procalcitonin were significantly higher in patients who died than in those who survived.

In our study, critically ill patients boarded in the ED for an average of 6.8 days (± 4.9) contributing to in-hospital mortality. This prolonged LOS can be attributed to several interrelated factors, many of which are deeply rooted in the constraints of the Lebanese healthcare system and the overwhelming burden on AUBMC. To begin with, EDs were operating at full capacity unable to accommodate the surge. AUBMC had indeed established a dedicated COVID-19 unit; however, it too was overloaded. The situation was further exacerbated by the fact that other hospitals in the region were also overwhelmed by the burden of their own patient influx and were unable to accept additional admissions.

One crucial moral obligation in this context was the duty to provide care to all individuals in need, and accordingly, the AUBMC could not decline care to incoming patients, which subsequently led to prolonged stays in the ED. Moreover, the catastrophic Beirut port explosion had devastating consequences on the region including the destruction of some major hospitals in Beirut. This placed an even greater burden and stress on AUBMC, as one of the largest tertiary centers in the area. The influx of patients from damaged or destroyed facilities added significantly to the challenges faced by the hospital. Our LOS is paralleled in multiple studies that showed a direct relation of mortality to the number of hours spent in the ED. A study by Singer et al. evaluated 41,256 admissions from the ED, and data presented that mortality increased from 2.5% in patients who boarded below 2 h to 4.5% in patients boarding 12 h or more (*p* < 0.001) [19]. Other studies similarly evaluated ED LOS association with mortality specifically on critically ill patients in ED, like Chaflin et al. who mentioned that ICU patients staying for more than 6 h in ED are 4.5% more likely to die (*p* value < 0.001). In addition, Al-Qahtani et al. revealed a significant increase in hospital mortality, 22.5%, compared with 29.1% and 37.2% in those boarding in the ED between 6 and 24 h or > 24 h, respectively [20, 21]. 51.9% of our patients who were labeled as dead in ED had boarded more than 6 h which comes in line with the high mortality rate observed in previous studies.

Nevertheless, few studies displayed that ED LOS has no effect on mortality [22, 23]. Only one study had a similar study population as ours, critically ill COVID-19 ED boarders [22]. Most of their patients had respiratory complications and only 8% had cardiac disease. Other studies mentioned that cardiac patients are prone to mortality certainly when there is a delay in time-sensitive interventions, such as initiation of mechanical ventilation, hemodynamic support, or monitoring, which might have a direct association with ED boarding time [24]. Our study supports this association since 42.8% of our patients were cardiac, and the second most common complication seen in our patients during their hospital stay was cardiac incidents (49%), which may have contributed to the higher mortality rate. We have reported a 65.5% mortality rate in contrast to the 22.4% reported by Ahlström et al., 32% by Alharthy et al., and 18% by Hu et al. To note in their studies, critically ill COVID patients were admitted directly to ICU units and did not board in the ED as ours [18–20].

Furthermore, our study highlighted in-hospital mortality predictors in line with other studies. Most of the patients who died were males and older than 65 years of age. A study conducted by Alharthy et al., in Saudi Arabia along with several other studies done in Sweden and China had demographic findings similar to ours [18–20]. Another study done by Zhao et al. have assessed the predictors of mortality in COVID ICU patients and have found that COPD, heart failure, pulse oxygen saturation, age (> 65), and smoking history were predictors of mortality in ICU patients [21]. Moreover, their results showed that patients who died had several comorbidities (smoking history, HTN, COPD, CAD, heart failure, and CKD) when compared to ICU patients who have survived [21]. These findings mirror ours since patients with several comorbidities were found to have an increased mortality. Patients who died were 1.5 times more likely to have cardiovascular diseases and 2.9 times more likely to have CKD, and twice more likely to have low oxygen saturation levels < 95% at triage.

As for COVID-19 treatments, our study highlighted the use of Actemra in COVID ICU patients. It resulted in significantly decreased mortality which is in line with a multicenter observational study that was done by Biran et al. in the USA which also showed that Actemra decreased mortality in COVID-19 patients who required ICU support. Besides, a systematic review revealed that Actemra probably reduces the mechanical ventilation rate, but our results could not be conclusive on the matter [22, 23]. The role of convalescent plasma in COVID ICU patients in terms of decreasing mortality is still controversial. Many reports stressed its role in decreasing mortality whereas other studies have found no clinical benefits of convalescent plasma in severe COVID-19 patients. Our results support that convalescent plasma does not significantly decrease mortality in COVID ICU patients [21, 24–26]. Hydroxychloroquine use did not decrease mortality in our COVID ICU patients, which is in line with several studies including the study by Lopez et al. which also showed that administering this drug to severely sick individuals has no added value in decreasing mortality and can increase the risk for QT prolongation [27–29]. A systematic review showed that Remdesivir and Ivermectin have an uncertain role in decreasing mortality and mechanical ventilation in COVID-19 patients [23]. In our study, Remdesivir and Ivermectin had no significant effects in either decreasing mortality or decreasing the need for intubation in COVID-19 ICU patients. The systematic review by Siemieniuk et al. has stated that the use of corticosteroids has moderately decreased mortality and mechanical ventilation in COVID-19 patients which was not seen in our study [23].

COVID-19 infection affects multiple organ systems leading to respiratory, cardiovascular, neurologic, gastrointestinal, and hematologic complications. Myocardial damage, myocarditis, acute myocardial infarction, heart failure, dysrhythmias, and venous thromboembolic events are common cardiovascular complications that have been reported in COVID-19 patients [30]. In our study, cardiovascular complications were significantly associated with higher mortality, and this was also seen in a study done in China where cardiac complications among hospitalized COVID-19 patients have resulted in an increased risk of in-hospital mortality [31]. Additionally, COVID-19-related acute respiratory distress syndrome (ARDS) mortality was reported to be significantly high in different populations, with the European population reporting the highest mortality in the world with an estimate of 73% in Poland [32]. Like other studies, our results showed a significantly increased mortality in COVID ICU patients who developed ARDS as a complication of their COVID-19 infection. Severe chest CT findings were significantly associated with mortality, which is in line with previous studies that had reported an increased mortality with a severe chest CT result [33]. These findings can guide emergency physicians and intensivists to predict a worse clinical outcome and start aggressive therapies early on in patients with severe COVID-19 imaging findings.

Previous publications correlated the severity of COVID-19 infection with high D-dimer levels, which was also demonstrated in our results, where 81.4% of our COVID-19 ICU boarders had abnormal D-dimer levels upon admission [34, 35]. A study by Ullah et al. had revealed that elevated D-dimer levels were significantly associated with higher in-hospital mortality which is in line with our results since higher D-dimer levels were associated with higher mortality but without significant results [35].

A meta-analysis that included 4631 COVID-19-infected individuals showed that elevated troponin levels were associated with an increased risk of severe illness, ICU hospitalization, and mortality. This is in line with our results where 39.1% of our patients had significantly elevated troponin levels [36].

In a single-centered study, in a non-ICU setting, non-invasive positive pressure ventilation (NPPV) was shown to be promising in COVID-19-associated acute hypoxemic respiratory failure [37]. However, this study was done in a non-ICU setting. Nevertheless, most guidelines (WHO, 2020, CCCGWG, 2020, NCCET, 2020, Indian CDC, 2020, NHS (NIV), 2020, ICSI, 2020) recommended against the use of non-invasive ventilation and encouraged other options like early intubation in critically ill patients such as patients with deteriorating respiratory status, hemodynamic instability, multiorgan failure, or aberrant mental condition [38]. Our study had no significant difference between patients who were on BiPAP and died versus those who survived. Almost 41.1% and 18.9% of those who were on BiPAP and high-flow nasal cannula died, respectively. However, patients who were on BiPAP were significantly less likely to get intubated.

A multi-centered study of 10 hospitals in the Chicago metropolitan area showed that age, sex (male), oxygen saturation, and history of diabetes are predictors of intubation [39]. In our study, although males, diabetics, and older people were more likely to get intubated, only patients who had an O_2_ < 95% at triage, those on vasopressors, dialysis, or those who received convalescent plasma, and those who developed respiratory complications were significantly more likely to be intubated.

A new organizational model was utilized in Italy, specifically the University Hospital of Pisa with the effort of multidisciplinary teams [40]. It insured a re-organization of emergency rooms to adapt to the emerging situation in terms of logistics [40]. After the activation of the hospital task force, measures were taken to face the outbreak [40]. Patients’ ED flow was constantly assessed, the same for hospitalization rate and COVID-19-related mortality [40]. They established a pre-triage setting in front of the ED to see suspected patients following designated criteria especially intensity of care [40]. Those patients were referred to specified areas for follow-up [40]. Patients’ evaluation was adjusted according to regional guidelines [40]. Tests and labs done allowed to classify patients in 4 levels of disease which helped stratify the risk of impending deteriorating patients [40]. Another model was designed for risk stratification using labs, imaging, and severity of disease to choose ventilator requirements and the needed level of care [41]. Eventually, to prevent patients from boarding a while in emergency rooms, they established VISUAL COVID, a new COVID bedding, monitoring management system to check for bed availability and moving patients fast to empty critical care units [40]. Effectively, lower mortality rates were observed, less hospitalizations rates, and reduction in patients’ flow [40]. Despite the challenging scenario, implementing new measures was successful [40]. Another university hospital in Italy, specifically Milan, also shared its adapted reorganization plan. This hospital split its facility into two separate ED entrances: the COVID-19 pathway and the non-COVID pathway [42]. Each of these had its own entrance, triage, and observation rooms [42]. Due to the ED overload of sick patients, the ward on the second floor was equipped and converted into a semi-intensive care unit to decrease the length of stay in the ED [42]. In another phase of the contingency plan, the waiting room was equipped with 14 oxygen nozzles for severe respiratory patients to buy time to transfer low-intensity patients and make room for the sick ones all while providing care [42]. In parallel in the USA, many hospitals worked to also find alternatives and decrease the burden on EDs. One healthcare system in all its 3 EDs implemented a plan to facilitate the workflow at the ED [43]. Focus on physical restructuring was first made and a similar pre-triage setting to that of Pisa, a tent, was constructed outside of the ED entrance to alleviate the load entering the ED [43]. To maximize staffing as much as possible, elective procedures and ambulatory visits were canceled leaving a pool of available providers to serve the COVID-19 patients [43]. In addition, a screening protocol was also adopted to identify patients at risk by standardizing a set of questions to be asked during ED triage [43].

This study has limitations related to its retrospective chart review nature. Even if we are a large tertiary care center and received a huge influx of COVID-19 patients, data was still collected from a single center. Despite these limitations, this study examined closely COVID ICU patients using a significant sample of patients and findings can be generalized.

## Conclusion

In conclusion, ICU boarders in the emergency department are the most vulnerable patients due to an increased risk of medical complications, which was noted in the COVID-19 era. Our study showed that 66% of our COVID ICU boarders died, 60% required intubation, and the majority developed complications. Age > 65 years old, vasopressor use, severe COVID CT chest findings, chemotherapy in the previous year, cardiovascular diseases, chronic kidney diseases, and low SaO_2_ < 95% on triage were risk factors significantly associated with mortality. Prolonged ED stay was specifically associated with increased mortality and in-hospital complications.

Emergency physicians and intensivists should be vigilant when taking care of ICU patients who board in the ED as they are a high-risk group prone to complications. Fragmented multiple teams care and the lack of a safe staffing model increases the risk of deterioration. All efforts need to be put to devise mitigation strategies and models of critical care delivery in the ED to alleviate the burden of ICU boarders and thus decrease morbidity and mortality. Lastly, we emphasize developing a well-structured protocol for the approach and management of patients, especially vulnerable populations who would board in emergency rooms during future pandemics. Lessons from this global health crisis should raise concern for complications seen in ED ICU boarders and allow the promotion of health measures optimizing resource allocation in future pandemic waves. A logistical re-organization of the ED is imperative.

## Data Availability

The datasets used and/or analyzed during the current study are available from the corresponding author on reasonable request.

## Abbreviations

ANC: Absolute neutrophil count
ARDS: Acute respiratory distress syndrome
AUBMC: American University of Beirut Medical Center
AUC: Area under the ROC curve
BiPAP: Bilevel positive airway pressure
BPE: Beirut port explosion
CAD: Coronary artery disease
CCCGWG: COVID-19 Clinical Care Guidance Working Group
CDC: Continuous discharge certificate
CI: Confidence interval
CKD: Chronic kidney disease
COPD: Chronic obstructive pulmonary disease
COVID-19: Coronavirus disease of 2019
CRP: C-reactive protein
CT: Computerized tomography
CVD: Cardiovascular disease
DIC: Disseminated intravascular coagulation
DM: Diabetes mellitus
ED: Emergency department
HTN: Hypertension
ICSI: Institute of Company Secretaries of India
ICU: Intensive care unit
IRB: Institutional review board
KM: Kaplan-Meier
LOS: Length of stay
NCCET: National Council for Continuing Education and Training
NHS: National Health Service
NIV: Non-invasive ventilation
NPPV: Non-invasive positive pressure ventilation
NY: New York
OR: Odds ratio
PCR: Polymerase chain reaction
PE: Pulmonary embolism
PT: Prothrombin time
ROC: Receiver operating characteristic
SPSS: Statistical Package for the Social Sciences
US: United States
WBC: White blood cells
WHO: World Health Organization
WI: Wisconsin
